# Serum Lipid Biomarkers and the Risk of Gastrointestinal Cancers in a Chinese Population: The Kailuan Prospective Study

**DOI:** 10.1002/cam4.70654

**Published:** 2025-02-06

**Authors:** Ying Xiao, Xin Du, Tianjie Wang, Dong Liu, Hongzhao You, Hao Wang, Hanyang Liang, Zhengqing Ba, Yilu Liu, Yu Ren, Jinghan Zeng, Weixian Yang, Shouling Wu, Jiansong Yuan

**Affiliations:** ^1^ Fuwai Hospital Chinese Academy of Medical Sciences and Peking Union Medical College, National Center for Cardiovascular Diseases Beijing China; ^2^ Department of Cardiology Kailuan General Hospital Tangshan China; ^3^ Peking Union Medical College Hospital Chinese Academy of Medical Sciences and Peking Union Medical College Beijing China

**Keywords:** cancer risk factors, colorectal cancer, digestive cancer, gastric cancer

## Abstract

**Background:**

Current evidence on relationships between serum lipid biomarkers and the risk of gastrointestinal cancers remains controversial, with no consensus reached.

**Methods:**

We conducted a prospective cohort study within the Kailuan Cohort wherein 88,225 individuals with baseline information on triglyceride (TG), total cholesterol (TC), low‐density lipoprotein cholesterol (LDL‐C), and high‐density lipoprotein cholesterol (HDL‐C) was followed from 2006 to 2021 for the incidence of esophageal cancer (EC), gastric cancer (GC), and colorectal cancer (CRC). Cox proportional hazards models and restricted cubic spline (RCS) analysis were used to estimate hazard ratios (HRs) and 95% confidence intervals (CIs).

**Results:**

Increased EC risk was associated with high HDL‐C levels (HR_Q4vs.Q1_ = 2.50, 95% CI: 1.57–3.98), while a U‐shaped relationship between HDL‐C and EC risk was revealed in the RCS analysis (*p*_overall_ ≤ 0.0001, *p*_nonlinear_ = 0.02). No robust association was identified between lipid biomarkers and GC risk. In multivariable analysis, increased CRC risk was positively associated with high TC levels (HR_Q4vs.Q1_ = 1.42, 95% CI: 1.11–1.83, *p*_trend_ = 0.03), dose–responsely negatively associated with LDL‐C levels over quartiles (HR_Q2vs.Q1_ = 0.83, 95% CI: 0.66–1.02; HR_Q3vs.Q1_ = 0.86, 95% CI: 0.69–1.07; HR_Q4vs.Q1_ = 0.68, 95% CI: 0.53–0.86, *p*_trend_ = 0.02), and showed a diminished negative association with HDL‐C levels over quartiles (HR_Q2vs.Q1_ = 0.75, 95% CI: 0.60–0.94; HR_Q3vs.Q1_ = 0.76, 95% CI: 0.61–0.95; HR_Q4vs.Q1_ = 0.91, 95% CI 0.74–1.13, *p*_trend_ = 0.02). The subsequent RCS analysis revealed a linear negative relationship of LDL‐C (*p*_overall_ = 0.004, *p*_nonlinear_ = 0.67) and a U‐shaped relationship of HDL‐C (*p*_overall_ = 0.05, *p*_nonlinear_ = 0.02) with CRC risk. Competitive risk analysis and sensitivity analysis confirmed the stability of our results.

**Conclusion:**

We observed a U‐shaped relationship regarding HDL‐C levels with EC and CRC risk, and a linear inverse relationship between LDL‐C levels and CRC risk. Relevant serum lipid levels should be properly managed in high‐risk individuals of certain gastrointestinal cancers.

## 1 Introduction

Gastrointestinal (GI) cancers, composed mainly of esophageal cancer (EC), gastric cancer (GC), and colorectal cancer (CRC), account for one‐fifth of new cancer cases and cause approximately 2.3 million deaths each year, according to Global Cancer Statistics 2020, imposing a tremendous social burden [1]. Primary prevention is of paramount importance to minimize the critical morbidity and mortality of GI cancers, among which identifying modifiable risk factors is a cornerstone.

In recent years, the deepening research on malignancy pathogenesis has made the association between metabolic factors and cancers a key focus [2]. Serum lipid biomarkers, notably triglyceride (TG), total cholesterol (TC), low‐density lipoprotein cholesterol (LDL‐C), and high‐density lipoprotein cholesterol (HDL‐C), serve as crucial clinical indicators of organic lipid metabolism. With an alarming prevalence rate exceeding one‐third in modern society [3], dyslipidemia has been implicated in numerous chronic diseases and stands as the established most significant risk factor for cardiovascular diseases (CVD) [4]. Dyslipidemia exhibits a high comorbidity rate with GI cancers [5].

There are multiple underlying biological mechanisms linking serum lipids to the carcinogenesis and progression of GI cancers, including inflammation, oxidative stress activation, cell signaling, immunomodulation, angiogenesis, and so on [6, 7]. Nevertheless, current evidence regarding associations between variability in lipid biomarkers and GI cancers yield highly disparate results among different lipid biomarkers, the same lipid biomarker across different GI cancers, and different studies, reporting positive [8, 9, 10, 11, 12], negative [9, 13, 14, 15], and non‐significant associations [16, 17, 18, 19, 20]. Such paradoxical findings pose challenges in defining a general lipid profile characteristic for the risk of GI cancers akin to that for CVD, leading to uncertainty in lipid management strategies for preventing GI cancers.

Conflicting findings in existing research may arise from small study sizes, short follow‐up periods, incomplete adjustment for potential confounders, lack of control for competing risks, insufficient consideration of exposure factors (focusing solely on a single lipid biomarker or treating dyslipidemia as an integral component of metabolic syndrome), the reverse effects of pre‐existing undetected cancers on serum lipid levels, and other potential biases [10, 11, 12, 21].

In light of the above, to address these uncertainties, we investigated the association between levels of major serum lipid biomarkers (TG, TC, LDL‐C, and HDL‐C) and GI cancer incidence based on a large Chinese prospective cohort.

## 2 Methods

### 2.1 Study Design and Population

The Kailuan Study is a prospective cohort study conducted within a community‐based population in Tangshan City, northern China (Trial Registration Number: ChiCTR‐TNRC‐11001489). Its primary purpose is identifying risk factors for chronic diseases, including cancer. Detailed study designs and procedures have been previously published [22, 23]. Briefly, 101,510 employees (81,110 males and 20,400 females, aged 18 years and above) from the Kailuan Group underwent biennial standard questionnaires, clinical and laboratory examinations at 11 affiliated hospitals from 2006 to 2007.

**FIGURE 1:**
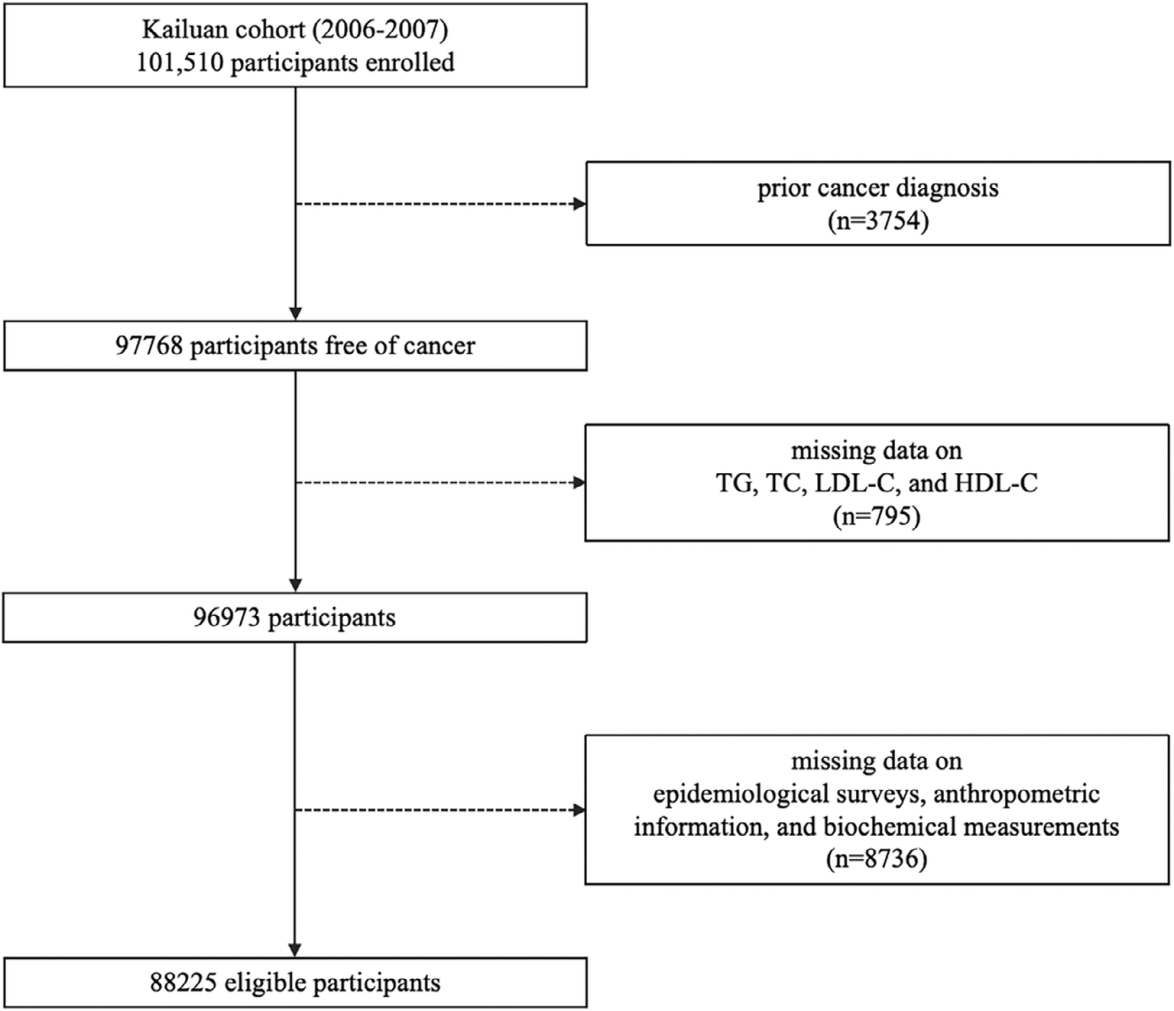
Flow chart of patient inclusion and exclusion.

In this study, participants were excluded based on the following criteria: (1) prior cancer diagnosis (*n* = 3754); (2) missing data on serum lipid biomarkers, including TG, TC, LDL‐C, and HDL‐C (*n* = 795); (3) missing information on potential confounders, including age, gender, fasting blood glucose (FBG), high‐sensitivity C‐reactive protein (hs‐CRP), body mass index (BMI), waist circumference (WC), hypertension, physical activity, smoking status, drinking status, and family cancer history (*n* = 8736). Finally, a total of 88,225 participants (70,715 males and 17,510 females) were enrolled (Figure 1). Additionally, participants developing GI cancers within 1 year after enrollment (*n* = 50), under lipid‐lowering therapies (LLT) (*n* = 908), and with high‐fat dietary habits (*n* = 7815) were further excluded separately in sensitivity analysis.

The study protocol conformed to the standards of the Declaration of Helsinki and was authorized by the Ethics Committees of Kailuan General Hospital. All participants provided written informed consent.

### 2.2 Measurement of Lipid Biomarkers

Blood samples were taken by trained phlebotomists after fasting overnight (8–12 h) using EDTA‐containing vacuum tubes, separated and kept at −80°C for further analysis. All blood samples underwent analysis at Kailuan General Hospital's central laboratory using an auto‐analyzer (Hitachi 747; Hitachi, Tokyo, Japan) to ensure the interassay coefficient of variation < 10% for each measurement. Serum lipid biomarkers, including TG, TC, LDL‐C, and HDL‐C, were measured using the enzymatic colorimetric method (Mind Bioengineering Co Ltd., Shanghai, China). Further details on the measurement process have been previously documented [24]. Serum lipid biomarker concentrations were categorized into quartiles based on the distribution in the overall population.

### 2.3 Measurement of Covariates

All baseline data were collected from participants during the first examination (2006–2007). Demographic and clinical characteristics, including age, gender, history of cancer, lipid‐lowering therapy, family cancer history, physical activity, smoking status, drinking status, and high‐fat dietary habits were collected via self‐reported information.

Biochemical parameters were obtained and analyzed as previously described. FBG was measured using the hexokinase/glucose‐6‐phosphate dehydrogenase method. hs‐CRP was measured using a high‐sensitivity nephelometry test (Cias Latex CRP‐H, Kanto Chemical Co., Tokyo, Japan).

Anthropometric data were obtained through clinical examinations conducted by trained medical assistants following standard operating procedures. The weight and height were measured on standard stadiometers and scales without wearing shoes. BMI was calculated by weight (kg)/height (m^2^). WC was measured at the midpoint between the upper plane of iliac crest and the lower edge of rib. Blood pressure (BP) was measured in the right upper arm using an electronic sphygmomanometer after 5 min of rest in a sitting position, with at least three measurements averaged. The diagnosis of hypertension was made if the participant: (i) had a previous history of hypertension; or (ii) had a systolic BP ≥ 140 mmHg and/or diastolic BP ≥ 90 mmHg; or (iii) was currently taking antihypertensive drugs.

### 2.4 Follow‐Up and Outcome Ascertainment

Person years of follow‐up were calculated from baseline until the date of cancer diagnosis, death, or termination of follow‐up (December 2021), whichever occurred first. Incident GI cancer cases were identified through self‐reporting every 2 years, medical linkage with the Tangshan medical insurance system and the Kailuan social security system, or discharge lists from the 11 affiliated hospitals. All cases underwent confirmation through medical records reviewed by clinical experts. GI cancers were coded according to the International Classification of Diseases (ICD), Tenth Revision. The site‐specific cancers included esophageal (C15), gastric (C16), and colorectal (C18–C20) cancers. Death certificates were also obtained annually from the Kailuan social security system. The Tangshan medical insurance and Kailuan social security system cover all participants' health information and living status.

### 2.5 Statistical Analysis

Mean ± standard deviation and one‐way analysis of variance (ANOVA) were utilized to describe and compare normally distributed variables, median (interquartile spacing) and nonparametric test (Kruskal‐Wallis H‐test) were used to describe and compare skewed distribution variables (TG and FBG). Absolute values with percentage and the chi‐square test were utilized to describe and compare categorical variables. Correlation tests were performed among lipid biomarkers (TG, TC, LDL‐C, HDL‐C as continuous variables) and covariates. Pearson correlation coefficient was calculated among normally distributed variables, while Spearman's rank correlation test was analyzed among skewed distribution and ranked variables.

Cox proportional hazard models were used to calculate hazard ratios (HR) and 95% confidence intervals (CI) between quartiles of each lipid biomarker (TG, TC, LDL‐C, and HDL‐C) relative to the reference group (1st quartile) and incidence of GI cancers. Multivariable analyses were adjusted for age, gender, FBG, hs‐CRP, BMI, WC, hypertension, physical activity, smoking status, drinking status, family cancer history, and other lipid biomarkers (e.g., TC, LDL‐C, and HDL‐C quartiles were adjusted in the analysis of TG variability). The above analyses were repeated using lipid biomarkers as continuous variables. The proportional hazard assumption was checked using Schoenfeld residual test. The restricted cubic spline (RCS) analysis with five knots at the 5th, 35th, 50th, 65th, and 95th percentiles of the distribution of each lipid biomarker was used in the multivariate model to explore dose–response relationships between lipid biomarkers and GI cancer risk.

Subgroup analyses were performed by FBG levels (< 7 mmol/L, and ≥ 7 mmol/L), age (< 45, 45–65, and ≥ 65 years), gender (male, female), BMI (< 24, 24–28, and ≥ 28 kg/m^2^), smoking status (non‐smoker, and former or current smoker), and drinking status (non‐drinker, and former or current drinker).

Sensitivity analysis was conducted to test the robustness of our findings. Firstly, the proportional subdistribution hazards model proposed by Fine and Gray [25] was used to consider competitive death events affecting GI cancer incidence. Secondly, GI cancer cases diagnosed within the 1st year of follow‐up were excluded to evaluate the possibility of reverse causation. Moreover, participants under lipid‐lowering therapy and those with regular high‐fat dietary habits were excluded separately to minimize the impact of medication and extreme dietary habits on serum lipid levels.

A *p*‐value (two‐sided) < 0.05 was considered statistically significant. Statistical analyses were performed using commercially available software (SAS software, version 9.4 and R software).

## 3 Results

### 3.1 Baseline Participant Characteristics

Baseline participant characteristics stratified by GI cancer sites are listed in Table 1. By December 2021, a total of 88,225 participants (80.15% men) were recruited in our study during a median follow‐up of 13.93 years, among which 190 (15.36%) cases of EC, 372 (30.07%) cases of GC, and 675 (54.57%) cases of CRC were diagnosed. The mean age in our entire population was 51.11 ± 12.49 years, while subjects with GC were significantly the oldest among all cancer groups (58.21 ± 10.24 years). Regarding baseline levels of lipid biomarkers, there were significant differences in the mean values of LDL‐C and HDL‐C as well as in the median levels of TG among different cancer sites, while TC levels did not differ significantly. Those who developed GC had the highest TG and LDL‐C levels of 1.32 (0.93, 2.02) mmol/L and 2.34 ± 0.87 mmol/L, respectively, while participants with CRC were found to have the lowest HDL‐C levels of 1.58 ± 0.45 mmol/L. Moreover, FBG, hs‐CRP, BMI, WC, hypertension, physical activity, smoking status, drinking status, family cancer history, and lipid‐lowering therapy differed significantly among different cancer groups.

**TABLE 1:** Baseline participant characteristics.

	All (*n* = 88,225)	Esophagus cancer (*n* = 190)	Gastric cancer (*n* = 372)	Colorectal cancer (*n* = 675)
Mean follow‐up time (year)	13.93 ± 3.00	8.32 ± 4.18	7.78 ± 4.50	8.28 ± 4.17
Age (year)	51.11 ± 12.49	55.01 ± 10.02	58.21 ± 10.24	57.24 ± 9.66
Male (%)	70,715 (80.15)	186 (97.89)	338 (90.86)	587 (86.96)
TG (mmol/L)	1.26 (0.89,1.90)	1.26 (0.90,1.96)	1.32 (0.93,2.02)	1.31 (0.95,1.92)
TC (mmol/L)	4.93 ± 1.14	5.06 ± 1.05	4.92 ± 1.21	5.02 ± 1.09
LDL‐C (mmol/L)	2.35 ± 0.87	2.32 ± 0.86	2.34 ± 0.87	2.26 ± 0.87
HDL‐C (mmol/L)	1.55 ± 0.40	1.76 ± 0.52	1.61 ± 0.45	1.58 ± 0.45
Hs‐CRP (mg/L)	0.87 (0.40,2.60)	0.90 (0.30,2.00)	0.92 (0.32,2.37)	0.98 (0.38,2.24)
BMI (Kg/m^2^)	24.98 ± 3.48	24.22 ± 3.39	25.00 ± 3.46	25.31 ± 3.48
WC (cm)	86.71 ± 9.96	88.14 ± 9.24	88.29 ± 9.59	88.48 ± 9.41
FBG (mmol/L)	5.11 (4.67,5.70)	5.19 (4.60,5.80)	5.21 (4.72,5.80)	5.16 (4.70,5.89)
Hypertension (%)	37,716 (42.75)	119 (62.63)	193 (51.88)	339 (50.22)
**Physical activity (%)**				
Never	7634 (8.65)	21 (11.05)	27 (7.26)	36 (5.33)
Occasionally	67,427 (76.43)	132 (69.47)	282 (75.81)	504 (74.67)
Regularly	13,164 (14.92)	37 (19.47)	63 (16.94)	135 (20.00)
**Smoking status (%)**				
Never	53,335 (60.45)	85 (44.74)	217 (58.33)	375 (55.56)
Past	4631 (5.25)	8 (4.21)	24 (6.45)	42 (6.22)
Moderate	3118 (3.53)	6 (3.16)	11 (2.96)	19 (2.81)
Severe	27,141 (30.76)	91 (47.89)	120 (32.26)	239 (35.41)
**Drinking status (%)**				
Never	52,660 (59.69)	82 (43.16)	233 (62.63)	367 (54.37)
Past	3157 (3.58)	10 (5.26)	13 (3.49)	24 (3.56)
Moderate	16,703 (18.93)	24 (12.63)	55 (14.78)	98 (14.52)
Severe	15,705 (17.80)	74 (38.95)	71 (19.09)	186 (27.56)
**High‐fat diet (%)**				
Seldom	7183 (8.14)	11 (5.79)	22 (5.91)	50 (7.41)
Occasionally	73,227 (83.00)	159 (83.68)	326 (87.63)	553 (81.93)
Regularly	7815 (8.86)	20 (10.53)	24 (6.45)	72 (10.67)
Lipid‐lowering therapy (%)	908 (1.03)	2 (1.05)	10 (2.69)	9 (1.33)
Cancer family history (%)	3093 (3.51)	6 (3.16)	25 (6.72)	22 (3.26)

*Note:* Data were shown as means ± standard deviation, median (interquartile spacing), or number (percentage). The bold values represent statistically significant differences in the analysis, specifically indicating a *p*‐value  <  0.05, meaning there is a significant difference between the groups.

Abbreviations: BMI, body mass index; FBG, fasting blood glucose; HDL‐C, high‐density lipoprotein cholesterol; Hs‐CRP, high‐sensitivity C‐reactive protein; LDL‐C, low‐density lipoprotein cholesterol; TC, total cholesterol; TG, triglyceride; WC, waist circumference.

**FIGURE 2:**
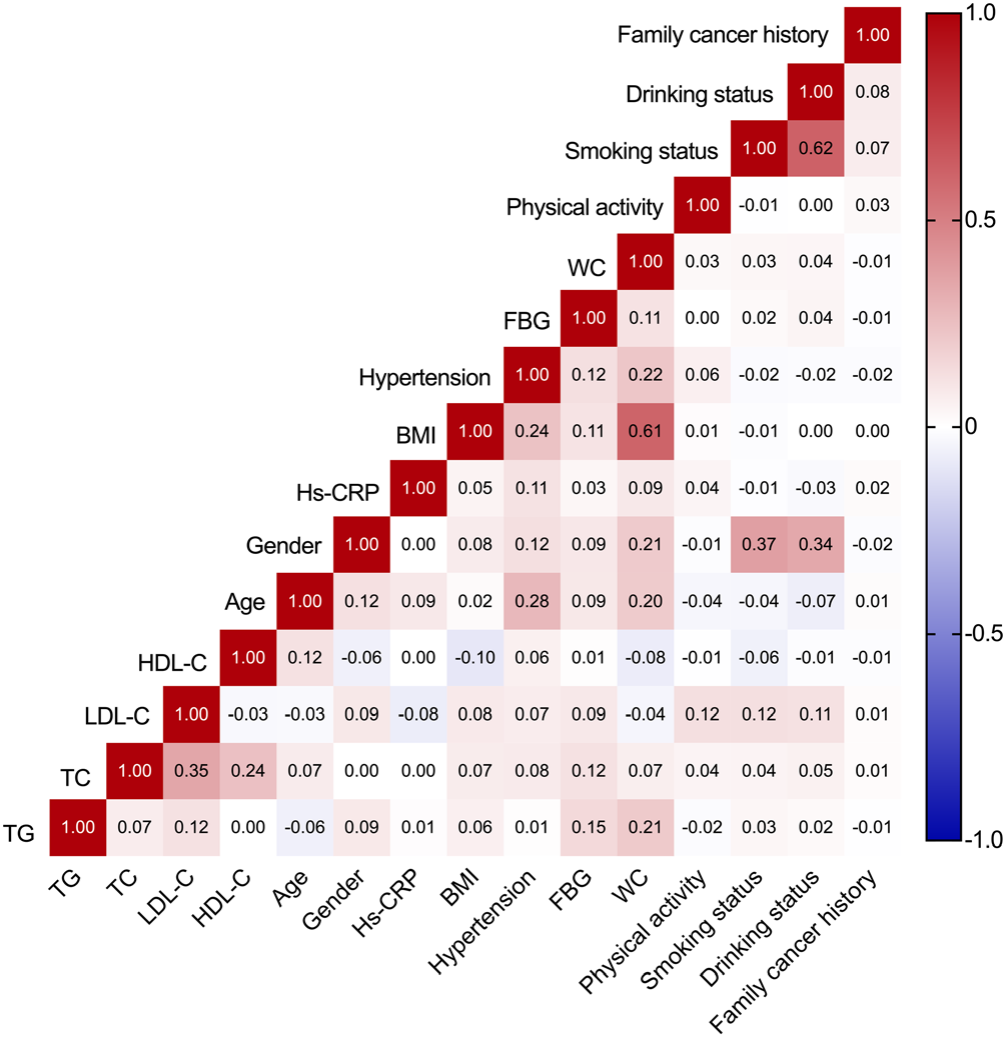
Correlation matrix of covariates. BMI, body mass index; FBG, fasting blood glucose; HDL‐C, high‐density lipoprotein cholesterol; Hs‐CRP, high‐sensitivity C‐reactive protein; LDL‐C, low‐density lipoprotein cholesterol; TC, total cholesterol; TG, triglyceride; WC, waist circumference.

In correlation analysis, weak to moderate correlations were observed among lipid biomarkers (*r* ranges from −0.029 between LDL‐C and HDL‐C to 0.347 between TC and LDL‐C, all *p* < 0.0001), and no strong correlation was detected in analysis among lipid indicators and other covariates (Figure 2).

### 3.2 Association Between Lipid Biomarkers and GI Cancer Risk

#### 3.2.1 Esophagus Cancer

Compared with the 1st quartile, HDL‐C levels over quartiles were observed to increase EC risk with a positive dose–response trend (crude HR_Q2vs.Q1_ = 1.11, 95% CI: 0.66–1.87; crude HR_Q3vs.Q1_ = 1.93, 95% CI: 1.20–3.08; crude HR_Q4vs.Q1_ = 3.07, 95% CI: 1.98–4.78; *p*_trend_ < 0.0001). Multivariable model further adjusted for age, gender, FBG, hs‐CRP, BMI, WC, hypertension, physical activity, smoking status, drinking status, family cancer history, and other lipid biomarkers slightly weakened but did not materially alter this positive trend (adjusted HR_Q2vs.Q1_ = 1.08, 95% CI: 0.64–1.84; adjusted HR_Q3vs.Q1_ = 1.83, 95% CI: 1.14–2.96; adjusted HR_Q4vs.Q1_ = 2.50, 95% CI: 1.57–3.98; *p*_trend_ < 0.0001). Such positive associations remained significant when HDL‐C was considered as a continuous variable (adjusted HR = 2.22, 95% CI: 1.71–2.89 per unit increment) (Table 2). Furthermore, RCS with 5 knots showed a salient nonlinear U‐shape association between HDL‐C and EC risk (*p*_overall_ < 0.0001, *p*_nonlinear_ = 0.02). With the 50th percentile of HDL‐C (1.51 mmol/L) chosen to be the reference, the HRs of EC related to HDL‐C levels increased sharply for HDL‐C levels less than 5th percentile (adjusted HR = 0.02; 95% CI: 0.00–0.87; 5th percentile cut‐off 0.99 mmol/L) or greater than 50th percentile (adjusted HR = 2.06; 95% CI: 1.45–2.93), while no significant association was discovered for HDL‐C levels between 5th to 50th percentile (Figure 4d). The highest TC levels were found to increase EC risk compared with the 1st quartile but only in the non‐adjusted model (crude HR_Q4vs.Q1_ = 1.71, 95% CI: 1.12–2.59). None of other lipid biomarkers showed significant associations with EC incidence (Table 2).

**TABLE 2:** Associations between lipid biomarkers (in quartiles and as continuous variables) and the risk of site‐specific gastrointestinal cancers.

	Esophagus cancer			Gastric cancer			Colorectal cancer		
Exposure	Cases (*n*)	Crude HR (95% CI)	Adjusted HR^a^ (95% CI)	Cases (*n*)	Crude HR (95% CI)	Adjusted HR^a^ (95% CI)	Cases (*n*)	Crude HR (95% CI)	Adjusted HR^a^ (95% CI)
**TG quartiles (mmol/L)**									
Q1 (< 0.89)	45	Ref.	Ref.	79	Ref.	Ref.	140	Ref.	Ref.
Q2 (0.89–1.26)	50	1.10 (0.74–1.65)	1.05 (0.70–1.58)	95	1.20 (0.89–1.61)	1.12 (0.83–1.52)	174	1.24 (0.99–1.54)	1.14 (0.91–1.42)
Q3 (1.26–1.90)	46	1.02 (0.68–1.54)	1.00 (0.65–1.54)	91	1.15 (0.85–1.55)	1.09 (0.80–1.50)	188	1.34 (1.08–1.67)	1.16 (0.93–1.46)
Q4 (≥ 1.90)	49	1.08 (0.72–1.62)	1.06 (0.69–1.64)	107	1.34 (1.01–1.80)	1.34 (0.98–1.83)	173	1.23 (0.98–1.53)	1.05 (0.82–1.33)
*p*_trend_		0.96	0.99		0.26	0.28		0.07	0.50
TG continuous (mmol/L)	190	1.01 (0.91–1.12)	0.99 (0.89–1.10)	372	1.05 (0.98–1.12)	1.06 (0.98–1.13)	675	1.01 (0.96–1.07)	0.99 (0.93–1.05)
*p* per 1 mmol/L increment		0.87	0.87		0.18	0.13		0.63	0.69
**TC quartiles (mmol/L)**									
Q1 (< 4.27)	35	Ref.	Ref.	100	Ref.	Ref.	135	Ref.	Ref.
Q2 (4.27–4.91)	48	1.38 (0.89–2.13)	1.25 (0.81–1.95)	93	0.93 (0.70–1.24)	0.86 (0.64–1.14)	161	1.20 (0.95–1.51)	1.16 (0.92–1.46)
Q3 (4.91–5.57)	47	1.33 (0.86–2.06)	1.12 (0.71–1.76)	75	0.74 (0.55–1.00)	0.64 (0.47–0.87)	181	1.33 (1.06–1.66)	1.33 (1.05–1.68)
Q4 (≥ 5.57)	60	1.71 (1.12–2.59)	1.31 (0.82–2.08)	104	1.03 (0.79–1.36)	0.81 (0.59–1.10)	198	1.46 (1.17–1.81)	1.42 (1.11–1.83)
*p*_trend_		0.09	0.64		0.14	0.05		0.007	0.03
TC continuous (mmol/L)	190	1.10 (0.98–1.24)	1.01 (0.89–1.15)	372	0.99 (0.91–1.09)	0.95 (0.87–1.04)	675	1.07 (1.00–1.14)	1.08 (1.00–1.15)
*p* per 1 mmol/L increment		0.10	0.85		0.90	0.29		0.04	0.04
**LDL‐C quartiles (mmol/L)**									
Q1 (< 1.84)	46	Ref.	Ref.	87	Ref.	Ref.	196	Ref.	Ref.
Q2 (1.84–2.34)	46	0.99 (0.66–1.49)	1.09 (0.71–1.67)	93	1.06 (0.79–1.42)	1.30 (0.96–1.75)	159	0.80 (0.65–0.99)	0.83 (0.66–1.02)
Q3 (2.34–2.83)	58	1.23 (0.84–1.82)	1.30 (0.86–1.97)	105	1.18 (0.89–1.57)	1.45 (1.08–1.96)	168	0.84 (0.68–1.03)	0.86 (0.69–1.07)
Q4 (≥ 2.83)	40	0.86 (0.57–1.32)	0.82 (0.51–1.33)	87	0.99 (0.74–1.33)	1.22 (0.87–1.70)	152	0.77 (0.62–0.95)	0.68 (0.53–0.86)
*p*_trend_		0.35	0.17		0.59	0.10		0.06	0.02
LDL‐C continuous (mmol/L)	190	0.96 (0.81–1.13)	0.95 (0.79–1.13)	372	0.98 (0.87–1.10)	1.03 (0.91–1.16)	675	0.87 (0.80–0.95)	0.84 (0.76–0.93)
*p* per 1 mmol/L increment		0.62	0.56		0.70	0.63		0.003	0.001
**HDL‐C quartiles (mmol/L)**									
Q1 (< 1.28)	26	Ref.	Ref.	86	Ref.	Ref.	175	Ref.	Ref.
Q2 (1.28–1.51)	30	1.11 (0.66–1.87)	1.08 (0.64–1.84)	85	0.95 (0.70–1.28)	0.97 (0.72–1.32)	142	0.80 (0.62–0.97)	0.75 (0.60–0.94)
Q3 (1.51–1.77)	52	1.93 (1.20–3.08)	1.83 (1.14–2.96)	84	0.94 (0.69–1.27)	0.96 (0.71–1.31)	150	0.82 (0.66–1.03)	0.76 (0.61–0.95)
Q4 (≥ 1.77)	82	3.07 (1.98–4.78)	2.50 (1.57–3.98)	117	1.32 (1.00–1.75)	1.27 (0.94–1.71)	208	1.16 (0.95–1.42)	0.91 (0.74–1.13)
*p*_trend_		< 0.0001	< 0.0001		0.04	0.17		0.001	0.02
HDL‐C continuous (mmol/L)	190	2.68 (2.10–3.42)	2.22 (1.71–2.89)	372	1.42 (1.12–1.79)	1.28 (1.00–1.63)	675	1.24 (1.03–1.49)	1.00 (0.83–1.21)
*p* per 1 mmol/L increment		< 0.0001	< 0.0001		0.004	0.05		0.02	0.10

Abbreviations: CI, hazard ratio; HDL‐C, high‐density lipoprotein cholesterol; HR, hazard ratio; LDL‐C, low‐density lipoprotein cholesterol; Q, quartile; TC, total cholesterol; TG, triglyceride.

^a^ Adjusted for age, gender, FBG, hs‐CRP, BMI, WC, hypertension, physical activity, smoking status, drinking status, family cancer history, and other lipid biomarkers (e.g., TC, LDL‐C, and HDL‐C were adjusted in the analysis of TG).

**FIGURE 3:**
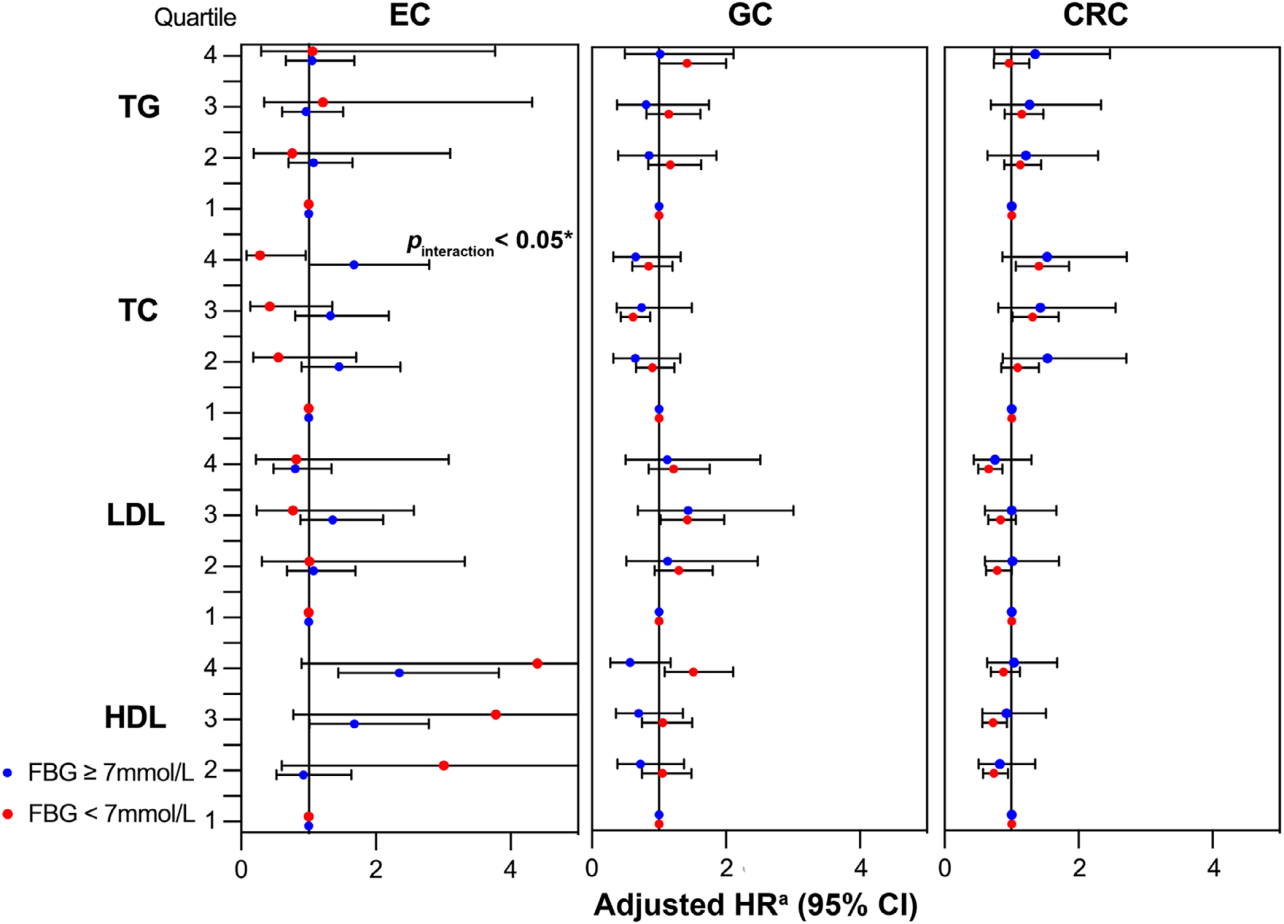
Associations of lipid biomarkers in quartiles with the risk of site‐specific gastrointestinal cancers by FBG subgroups. CI, hazard ratio; FBG, fasting blood glucose; HDL‐C, high‐density lipoprotein cholesterol; HR, hazard ratio; LDL‐C, low‐density lipoprotein cholesterol; TC, total cholesterol; TG, triglyceride. ^a^Adjusted for age, gender, hs‐CRP, BMI, WC, hypertension, physical activity, smoking status, drinking status, family cancer history, and other lipid biomarkers (e.g., TC, LDL‐C, and HDL‐C were adjusted in the analysis of TG). *p_interaction_ < 0.05, statistically insignificant *p*_interaction_ in subgroup analysis was omitted.

Subgroup analyses by FBG, age, gender, BMI, smoking habits, and drinking habits revealed no significant interaction between lipid biomarkers and EC risk, with the exception of FBG and TC (*p*_interaction_ < 0.05) (Figure 3 and Figures S1–S5). Grouping by FBG levels resulted in a positive association between high TC levels and EC risk in FBG ≥ 7 mmol/L group (adjusted HR_Q4vs.Q1_ = 1.67, 95% CI: 1.01–2.79) but a reverse negative association in contrast in FBG < 7 mmol/L group (adjusted HR_Q4vs.Q1_ = 0.28, 95% CI: 0.08–0.96) (Figure 3).

#### 3.2.2 Gastric Cancer

**FIGURE 4:**
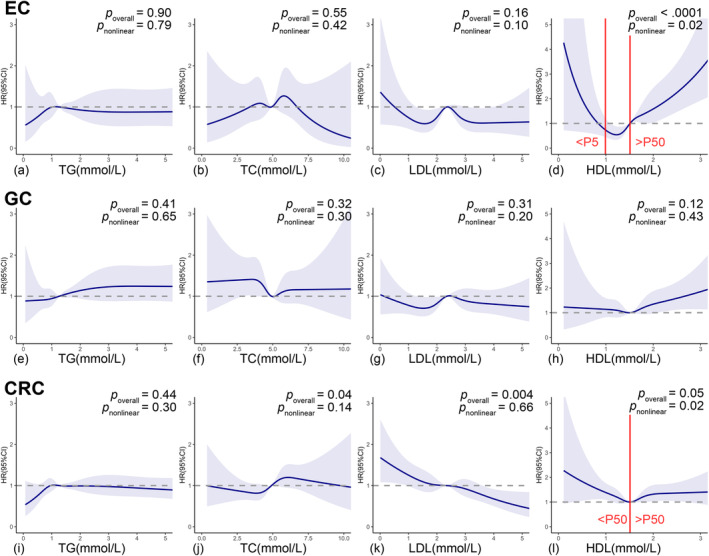
RCS curves for the association of lipid biomarkers with the risk of site‐specific gastrointestinal cancers. CI, hazard ratio; CRC, colorectal cancer; EC, esophageal cancer; FBG, fasting blood glucose; GC, gastric cancer; HDL‐C, high‐density lipoprotein cholesterol; HR, hazard ratio; LDL‐C, low‐density lipoprotein cholesterol; TC, total cholesterol; TG, triglyceride. Cubic spline graph for the association of TG, TC, LDL‐C, and HDL‐C with the risk of EC (a–d), GC (e–h), and CRC (i–l). Reference: 50th percentile; Knots: 5th, 35th, 50th, 65th, and 95th percentile; Adjusted HR: The solid line; 95% CI: The shaded area. Adjusted for age, gender, FBG, hs‐CRP, BMI, WC, hypertension, physical activity, smoking status, drinking status, family cancer history, and other lipid biomarkers (e.g., TC, LDL‐C, and HDL‐C were adjusted in the analysis of TG).

In Table 2, Non‐adjusted model discovered borderline positive associations between GC incidence and the highest TG and HDL‐C levels compared with the lowest quartile, with corresponding crude HRs of 1.34 (95% CI: 1.01–1.80) and 1.32 (95% CI: 1.00–1.75), respectively. Nevertheless, such association diminished to insignificant when further adjusted for potential confounders. The 3rd quartile of TC levels was found to be associated with decreased GC risk compared with the 1st quartile (crude HR_Q3vs.Q1_ = 0.74, 95% CI: 0.55–1.00) and this association remained with a borderline significant negative trend over quartiles in multivariable model (adjusted HR_Q2vs.Q1_ = 0.86, 95% CI: 0.64–1.14; adjusted HR_Q3vs.Q1_ = 0.64, 95% CI: 0.47–0.87; adjusted HR_Q4vs.Q1_ = 0.81, 95% CI: 0.59–1.10, *p*_trend_ = 0.05). Multivariable model also resulted in significant positive association between the 3rd quartile LDL‐C levels and GC risk compared with the 1st quartile (adjusted HR_Q3vs.Q1_ = 1.45, 95% CI: 1.08–1.96). When analyzing lipid biomarkers as continuous variables, only a borderline positive association between HDL‐C and GC risk was discovered (adjusted HR = 1.28, 95% CI: 1.00–1.63 per unit increment). Nevertheless, despite the above associations identified in Cox proportional hazard models, subsequent RCS analysis detected no significant linear or nonlinear relationship between any lipid biomarker and GC incidence (Figure 4e–h).

The above results remained similar in different FBG, age, gender, BMI, smoking and drinking habit subgroups (all *p*_interaction_ > 0.05) (Figure 3, and Figures S1–S5).

#### 3.2.3 Colorectal Cancer

In non‐adjusted model, significant positive associations between CRC risk and TC were pronounced over quartiles with a clear dose–response trend (crude HR_Q2vs.Q1_ = 1.20, 95% CI: 0.95–1.51; crude HRQ3vs.Q1 = 1.33, 95% CI: 1.06–1.66; crude HR_Q4vs.Q1_ = 1.46, 95% CI: 1.17–1.81, *p*_trend_ = 0.007). In multivariable model, such positive associations maintained significant over TC quartiles (adjusted HR_Q2vs.Q1_ = 1.16, 95% CI: 0.92–1.46; adjusted HR_Q3vs.Q1_ = 1.33, 95% CI 1.05–1.68; adjusted HR_Q4vs.Q1_ = 1.42, 95% CI: 1.11–1.83, *p*_trend_ = 0.03) but diminished to borderline when analyzing TC as continuous variable (adjusted HR =1.08, 95% CI 1.00–1.15 per unit increment) (Table 2). Correspondingly, the cubic spline curve for TC and CRC risk was a vertical line approximately parallel to the X‐axis (*p*_overall_ = 0.04, *p*_nonlinear_ = 0.15) (Figure 4j). Subgroup analyses were performed within different FBG, age, gender, BMI, smoking and drinking habits groups (Figure 3 and Figures S1–S5), among which only age significantly modified the association between TC in quartiles and CRC risk (*p*_interaction_ < 0.05) (Figure S2). Elevated levels of TC were associated with increased CRC risk only in young (age < 45 years) and middle‐aged groups (age 45–65 years).

Low‐density lipoprotein cholesterol showed a sound dose–response negative association with CRC risk analyzed either in quartiles or as a continuous variable in both crude model (In quartiles, crude HR_Q2vs.Q1_ = 0.80, 95% CI: 0.65–0.99; crude HR_Q3vs.Q1_ = 0.84, 95% CI; 0.68–1.03; crude HR_Q4vs.Q1_ = 0.77, 95% CI: 0.62–0.95, *p*_trend_ = 0.06; In per unit increment, crude HR = 0.87, 95% CI: 0.80–0.95) and multivariable model (In quartiles, adjusted HR_Q2vs.Q1_ = 0.83, 95% CI: 0.66–1.02; adjusted HR_Q3vs.Q1_ = 0.86, 95% CI: 0.69–1.07; adjusted HR_Q4vs.Q1_ = 0.68, 95% CI: 0.53–0.86, *p*_trend_ = 0.02; In per unit increment, adjusted HR = 0.84, 95% CI: 0.76–0.93) (Table 2). Consistently, a linear negative association was confirmed in the RCS model (*p*_overall_ = 0.004, *p*_nonlinear_ = 0.67) (Figure 4k). Subgroup analyses did not alter this negative association (all *p*_interaction_ > 0.05) (Figure 3 and Figures S1–S5).

Compared with the 1st quartile, the 2nd quartile of HDL‐C was observed to decrease the risk of CRC by 20% (Q2 vs. Q1, 95% CI: 0.65–0.99). This negative association attenuated to insignificantly 18% in the 3rd quartile (Q3 vs. Q1, 95% CI: 0.66–1.03) and reversed to insignificantly increase CRC risk by 16% in the highest quartile (Q4 vs. Q1, 95% CI: 0.95–1.42; *p*_trend_ = 0.0001). Similarly, a diminished negative trend between HDL levels over quartiles and CRC risk could be witnessed in multivariable model (adjusted HR_Q2vs.Q1_ = 0.75, 95% CI: 0.60–0.94; adjusted HR_Q3vs.Q1_ = 0.76, 95% CI: 0.61–0.95; adjusted HR_Q4vs.Q1_ = 0.91, 95% CI: 0.74–1.13, *p*_trend_ = 0.02). Noteworthy, a contradictory positive association was identified when analyzing HDL‐C as a continuous variable (crude HR = 1.24, 95% CI: 1.03–1.49 per unit increase), although attenuated to insignificant after adjusting for potential confounders (Table 2). RCS analysis showed a U‐shape nonlinear relationship between HDL‐C and CRC incidence (*p*_overall_ = 0.05, *p*_nonlinear_ = 0.02), which might explain above conflicting results: With the 50th percentile of HDL‐C (1.51 mmol/L) chosen to be the reference, the HRs of CRC decreased with the rise of HDL‐C concentrations for HDL‐C less than 50th percentile (adjusted HR = 0.50, 95% CI: 0.59–0.86) but slowly increase with the rise of HDL‐C concentrations while HDL‐C exceed 50th percentile (adjusted HR = 1.25; 95% CI 0.93–1.66) (Figure 4l). The above results did not differ in age, gender, FBG, BMI, drinking and smoking subgroups (all *p*_interaction_ > 0.05) (Figure 3 and Figures S1–S5).

As for TG, survival analysis detected a positive association between the 3rd quartile compared with the 1st quartile in non‐adjusted model (crude HR_Q3vs.Q1_ = 1.34, 95% CI: 1.08–1.67) (Table 2), which was found to exist only in non‐drinkers in subsequent subgroup analyses (*p*_interaction_ < 0.05) (Figure 3 and Figures S1–S5).

### 3.3 Sensitivity Analysis

In sensitivity analysis, our main findings were not materially altered after further consideration of competitive events (Table S1), excluding participants who diagnosed GI cancers within the 1st year of follow‐up (*n* = 7 for EC, *n* = 17 for GC, *n* = 26 for CRC), under lipid‐lowering therapies (*n* = 908), and with regular high‐fat dietary habits (*n* = 7815) (Tables S2–S4).

## 4 Discussion

In this prospective study of a large Northern Chinese population cohort, we investigated the association between prime lipid biomarkers and site‐specific GI cancers. We found a salient U‐shape relationship between HDL‐C levels and EC incidence, with excessive EC risk for high and extremely low HDL‐C levels. In subgroup analysis, TC was found to be positively associated with EC risk in the abnormal FBG group, whereas it was found to be inversely negatively associated with EC risk in the normal FBG group. No robust association was identified between prime lipid biomarkers and GC risk. LDL‐C and HDL‐C levels demonstrated a linear negative relationship and a nonlinear U‐shape relationship with CRC risk, respectively. Participants, especially young and middle‐aged groups with high TC levels, exhibited a higher risk of CRC incidence. Competitive risk analysis and sensitivity analysis confirmed the robustness of our results. To our knowledge, this report for the first time revealed the U‐shape hazard curve of HDL‐C in EC and CRC, offering new perspectives on lipid management strategies for GI cancer prevention.

The pathogenesis of GI cancers is a complex, multifactorial process involving genetic and environmental factors. Existing epidemiological studies on the association between lipid biomarkers and incidence of malignancies have yielded highly divergent results. Such inconsistency may be attributed to the intricate bidirectional influences of serum lipids on carcinogenesis, suggesting the varied roles serum lipid plays among different cancer types and highlighting the necessity of tumor‐specific analysis.

In the pro‐oncogenic aspect, hypercholesterolemia and hypertriglyceridemia can intensify inflammation and oxidative stress, fostering a mutagenic inflammatory microenvironment and excessive ROS/RNS, thereby leading to DNA damage, activating proto‐oncogenes (e.g., Ras) and suppressing tumor suppressor genes (e.g., P53) [6]. Lipid rafts on the cell membrane transmit multiple oncogenic signals, and elevated cholesterol levels in lipid rafts was proven to activate several pro‐oncogenic pathways (e.g., Hedgehog and IL6‐STAT3 pathways) [26]. Furthermore, heightened serum TC can also accelerate epithelial‐mesenchymal transition, promote immunosuppression and angiogenesis, and increase blood viscosity, collectively promoting tumorigenesis, progression, and metastasis [6]. Several cholesterol metabolites, such as bile acids, act as carcinogenic stimulants in the progression of precancerous GI lesions [26]. In addition, hypertriglyceridemia acts as a component of metabolic syndrome and closely correlates with glucometabolic disorders and insulin resistance (IR). IR leads to elevated insulin‐like growth factor (IGF) levels, activating the IGF‐1/IGF‐1R signaling pathway. This pathway regulates the cell cycle and activates Ras/MAPK, PI3K, mTOR, and AKT pathways, promoting abnormal cell proliferation and malignant transformation [6].

In the anti‐oncogenic aspect, however, there also remain substantial underlying mechanisms [26, 27, 28]. Firstly, serum lipids and their metabolites demonstrate bidirectional immunomodulatory effects, potentially exerting anti‐tumor effects through positive immune modulation. Cholesterol, for instance, has been shown to augment the number and activity of CD8+ T cells [27] and induce the secretion of various cytokines (e.g., IFN‐γ) in NK cells through cell membrane signaling or acting as structural components [28]. Elevated levels of TG enhance the cross‐presentation ability of dendritic cells, while fatty acids serve as a crucial fuel source to sustain the anti‐tumor activity of diverse immune cells [28]. Secondly, specific lipid metabolites, such as DDA, exhibit anti‐tumor activity and manifest potent anticancer effects across various cancer types [26]. It is noteworthy that reverse causation is suggested as another explanation for negative correlations. Undiagnosed early‐stage cancers might deplete lipids, acting as a potential confounder between lipid levels and cancer risk [18, 29].

### 4.1 Lipid Biomarkers and EC Risk

Evidence on HDL‐C in relation to EC incidence was limited to two studies, one of which categorized HDL‐C concentration as “high” or “low” and reported insignificant results with esophageal adenocarcinoma and esophageal squamous‐cell carcinoma risk based on a Norway cohort [30]. Another Swedish AMORIS study discovered a positive relationship between EC risk and high HDL‐C concentration [9]. This positive association was consistent and more pronounced in our study, with the highest quartiles increasing EC risk by significantly 150%, uninfluenced by subgroup or sensitivity analysis. A step further, we probed a prominent reverse association of EC incidence with HDL‐C decline in extremely low levels (< 5 percentile).

Most previous studies failed to find a significant relationship between TG, TC, LDL‐C and EC risk [16, 30]. Our findings regarding TG and LDL‐C levels with EC risk are consistent with prior perspectives. However, we found interestingly opposing positive and negative associations between TC and EC risk in abnormal and normal FBG subgroups. This discovery implies that the pathogenesis of high TC‐promoted EC carcinogenesis may be mediated through aberrant glucose metabolism, further investigation is warranted to elaborate on detailed underlying metabolic mechanisms of EC carcinogenesis and refine risk stratification.

### 4.2 Lipid Biomarkers and GC Risk

Our findings on GC generally align with many previous studies, identifying no robust associations among lipid biomarkers [9, 10, 18, 31]. Our study discovered a borderline weak negative trend between TC over quartiles and GC risk. Such association is more pronounced in the other two Japanese cohorts, JPHC and Hisayama studies [13, 14]. The observed negative associations can be attributed to Japanese traditional low‐fat dietary patterns, small sample sizes, lack of adjustment to lipid‐lowering therapy, and the possibility of reverse causation.

Additionally, it is worth noting that a few studies have suggested a negative correlation between serum HDL‐C levels and GC risk [32, 33, 34]. However, four well‐designed Mendelian randomization studies—an advanced epidemiological method that uses genetic variants to assess causal relationships while minimizing confounding and reverse causation—have confirmed that HDL‐C and its apolipoprotein component ApoA1, are not associated with GC risk in different country and ethnic groups [35, 36, 37, 38]. A potential explanation for these conflicting findings is that *Helicobacter pylori* infection, a significant risk factor for GC, can lead to a marked subsequent decrease in circulating HDL‐C levels [39]. Moreover, as GC progression can also result in the depletion of various lipid levels, including HDL‐C [40], we suggest that the observed negative correlation in these studies might be due to a lack of adjustment for *Helicobacter pylori* infection status and reverse causation [34]. Further genomics studies and laboratory research are needed to clarify the causal relationship between HDL‐C and GC risk.

### 4.3 Lipid Biomarkers and CRC Risk

Among GI cancers, associations between serum lipids and CRC are the tightest and most widely studied. Hypercholesterolemia is a relatively recognized risk event for CRC, which has been demonstrated in several Mendelian randomization studies [41], meta‐analyses [12, 42], and multiple large cohorts [9, 18, 43, 44]. Nevertheless, there are also a few studies that have reported no association [21, 45]. Our findings reported a borderline positive association, with per 1 mmol/L increment of TC increasing CRC risk by around 8%.

There are conflicting studies on TG advocating either a positive [9, 12, 41, 42, 43] or an insignificant [41, 44, 45] association with CRC risk. Our findings between hypertriglyceridemia and CRC risk are generally insignificant.

We observed a salient linear negative dose–response relationship between LDL‐C and CRC risk. This finding is consistent with several former studies, although most results were insignificant [9, 45, 46], among which a previously published study also based on the Kailuan cohort reported that increasing LDL‐C levels were associated with decreased total cancer risk, and insignificantly decreased CRC risk [47]. Notably, the negative association was intensified in that study even after excluding the first 4 years' new‐onset cancer cases, reducing the likelihood of reverse causation. Mechanistically, this could be linked to the anti‐oncogenic, immunomodulatory role of LDL‐C's cholesterol components, as previously mentioned, suggesting that LDL‐C may play a potential protective role in CRC development [27, 28]. However, conflicting results have also been reported in other studies [42, 44], indicating that the role of LDL‐C in CRC carcinogenesis may be sophisticated. This underscores the need for further research to gain a more thorough understanding of the underlying mechanisms involved.

Regarding HDL‐C and CRC risk, limited studies reported highly varied results [12, 15, 41, 42, 48]. The Women's Health study concerning long‐term cancer risk reported that the highest quartile of HDL‐C decreases CRC risk significantly by 37% [48]. However, Yang, M.H., et al. claimed an opposite 12% higher CRC risk of highest HDL levels compared with the lowest in their large cross‐sectional study [15]. Two meta‐analyses and a Mendelian randomization reported no association [12, 41, 42]. Noteworthy, none of the above studies have studied the possible nonlinear association. Our study for the first time discovered a U‐shape nonlinear relationship between HDL‐C and CRC risk with the median (1.51 mmol/L) as the inflection point, providing a new interpretation of prior contradictory results.

### 4.4 The U‐Shaped Association Between HDL‐C and the Risk of EC and CRC

The U‐shaped association between cancer mortality and HDL‐C, or its components ApoA1, has been explored by several studies in recent years [49, 50]. For example, a recent finding by Morland J.G. et al. in a large prospective cohort with a follow‐up period of 16.8 years had discovered a clear U‐shaped relationship between HDL‐C and total cancer mortality. Subsequent subgroup analyses of site‐specific cancers reveal that HDL‐C was U‐shapedly associated with CRC mortality [49]. However, similar associations with cancer incidence have not yet been reported, and our study is the first to identify a U‐shaped association between serum levels and the incidence of EC and CRC, providing important clinical evidence for understanding the bidirectional role of HDL‐C in cancer pathophysiology.

On the one hand, often hailed as the “good cholesterol”, HDL‐C demonstrates antioxidant, anti‐inflammatory, immunostimulatory, anti‐apoptotic, and anti‐thrombotic functions, either indirectly through reverse cholesterol transport or directly, attributing to its potential for anti‐tumor effects [26, 51]. On the other hand, the structure and distribution of HDL‐C particles are crucial to their function, and alterations in these particles may contribute to carcinogenic processes [51]. For instance, ApoM, a subsidiary lipoprotein particle of HDL‐C, can interact with SIP and trigger the sphingosine kinase 1 (Sphk1)/SIP signaling pathway, whose activation of the downstream IL‐6/STAT3/Akt pathway is implicated in CRC carcinogenesis [51]. Therefore, the increased risk of EC and CRC associated with high HDL‐C levels may be due to modifications in its particle composition. Furthermore, IR, a key metabolic mechanism in EC and CRC carcinogenesis, may serve as an important link [52]. Under IR conditions, HDL‐C particles undergo characteristic changes, becoming triglyceride‐rich and cholesterol‐depleted, which leads to impaired antioxidant capacity [26]. In vitro studies have also shown that oxidized HDL‐C can promote breast cancer metastasis [53]. Collectively, further research into the quantity, composition, and function of HDL‐C particles may offer valuable insights into the role of lipid metabolism in EC and CRC carcinogenesis.

Moreover, even within the field of CVD, epidemiological studies show that extremely high levels of HDL‐C appear to lose their protective benefits [54, 55]. A large Copenhagen prospective study found a U‐shaped relationship with CVD mortality. In the extremely high HDL‐C group (≥ 3.00 mmol/L in men and ≥ 3.50 mmol/L in women), the risk of CVD mortality increased by 2–3 times compared to the reference group (1.50–1.99 mmol/L in men and 2.00–2.49 mmol/L in women). A similar U‐shaped relationship was also observed for all‐cause mortality [54]. Notably, the inflection point of HDL‐C levels associated with EC and CRC risk in our study (1.51 mmol/L) was lower compared to studies concerning CVD mortality (1.78–2.00 mmol/L) [54, 55], possibly due to differences in the pathogenic mechanisms of HDL‐C in CVD versus oncology [56].

Together with accumulating evidence from various fields, the traditional view of HDL‐C as purely “good cholesterol” seems oversimplified [56]. Future large cohort studies and randomized controlled trials are essential to determine the optimal HDL‐C concentration range that maximizes overall benefits in controlling CVD events and cancer risk.

Our study has some prominent strengths. Firstly, to our knowledge, this is the largest prospective Asian cohort evaluating the association between lipid biomarkers and GI cancers, with a long follow‐up duration reducing the potential impact of reverse causation. Secondly, all participants in Kailuan Study are employees whose expenses were fully covered. Outcome information was acquired from the Tangshan Medical Insurance System and the Kailuan Social Security System. Thereby, the study achieved an almost 100% follow‐up rate. Thirdly, we obtained information of serum lipid biomarkers via well‐standardized measurements and made relatively comprehensive adjustments of potential confounders. Moreover, sensitivity analysis was performed to further adjust competitive risk, minimize reverse causation bias, reduce direct effects of high‐fat diet on GI tract and impact of lipid‐lowering therapy on serum lipids.

Nevertheless, the limitations of our study should not be neglected. Firstly, the vast majority of Kailuan study participants are male, such gender imbalance requires more caution when interpreting our findings in females. Secondly, serum lipid biomarkers, only measured at baseline, might not reflect the potential fluctuation in concentrations during the study period. However, the influence is assumed to be minor as lipid markers have been shown to be quite stable over multiple years [57]. Thirdly, due to the lack of information, we were unable to adjust for several precancerous conditions such as colorectal polyps and adenomas, which may potentially affect serum lipid levels [42]. Colon cancer and rectal cancer could not be analyzed separately due to the same reason, which prevents us from discussing the separate effects of serum lipids on colon and rectal cancers.

## 5 Conclusions

In this large prospective cohort study in a Chinese population, we identified a U‐shaped relationship between serum HDL‐C levels and the incidence of EC and CRC, along with a negative dose–response association between LDL‐C revels and CRC risk. Our findings advocate for a more prudent approach to manage serum lipid levels in high‐risk EC and CRC patients. Health checkups should consider serum lipid abnormalities as potential risk factors for certain GI cancers. Future research should focus on: (1) elucidating the underlying mechanisms by which serum lipid levels influence GI carcinogenesis; (2) conducting gender‐balanced multicenter large randomized controlled trials to confirm the causal relationship between serum lipid levels and GI cancer risk; (3) implementing long‐term and large‐scale cohort studies that track both GI cancer incidence and major adverse cardiovascular events, which will help developing integrated lipid management strategies that balance the “benefits” of reducing GI cancer risk with the potential “risk” of increasing CVD risk.

## Author Contributions

**Ying Xiao:** conceptualization (lead), data curation (lead), formal analysis (lead), investigation (lead), methodology (equal), validation (lead), visualization (equal), writing – original draft (lead). **Xin Du:** data curation (equal), formal analysis (equal), methodology (lead), visualization (equal), writing – original draft (equal). **Tianjie Wang:** formal analysis (equal), investigation (equal), validation (equal). **Dong Liu:** formal analysis (equal), validation (equal), visualization (equal). **Hongzhao You:** formal analysis (equal), validation (equal), visualization (equal). **Hao Wang:** validation (equal), visualization (equal). **Hanyang Liang:** validation (equal), visualization (equal). **Zhengqing Ba:** visualization (equal). **Yilu Liu:** visualization (equal). **Yu Ren:** visualization (equal). **Jinghan Zeng:** investigation (equal). **Weixian Yang:** methodology (equal), supervision (equal), writing – review and editing (equal). **Shouling Wu:** methodology (equal), resources (lead), supervision (equal), writing – review and editing (supporting). **Jiansong Yuan:** methodology (lead), supervision (lead), writing – review and editing (lead).

## Ethics Statement

The studies involving human participants were reviewed and approved by the ethic committee of the Kailuan General Hospital. The participants provided their written informed consent to participate in this study.

## Conflicts of Interest

The authors declare no conflicts of interest.

## Supporting information


Data S1.

## Data Availability

The datasets for this manuscript are not publicly available because all our data are under regulation of both the National Cancer Center of China and Kailuan Group. Requests to access the datasets should be directed to SLW, drwusl@163.com.
