# Supplementary material for: Serum Lipid Biomarkers and the Risk of Gastrointestinal Cancers in a Chinese Population: The Kailuan Prospective Study
Source: Cancer Med. 2025 Feb 6;14(3):e70654. doi: 10.1002/cam4.70654 (PMC11799922; doi:10.1002/cam4.70654)
Supplement: Supplementary file 1 — Data S1. [file CAM4-14-e70654-s001.docx]

**Table S1** Competitive risk analysis for associations of lipid biomarkers (in quartiles and as continuous variables) with the risk of site-specific gastrointestinal cancers.

| Exposure | Esophagus cancer | Gastric cancer | Colorectal cancer |
| --- | --- | --- | --- |
|  | Adjusted HR^a^ (95%CI) | Adjusted HR^a^ (95%CI) | Adjusted HR^a^ (95%CI) |
| TG quartiles (mmol/L) |  |  |  |
| Q1 (<0.89) | Ref. | Ref. | Ref. |
| Q2 (0.89-1.26) | 1.04 (0.70-1.57) | 1.12 (0.83-1.53) | 1.14 (0.91-1.43) |
| Q3 (1.26-1.90) | 0.99 (0.64-1.54) | 1.09 (0.80-1.50) | 1.17 (0.93-1.47) |
| Q4 (≥1.90) | 1.05 (0.67-1.65) | 1.34 (0.98-1.84) | 1.05 (0.82-1.34) |
| *p*_trend_ | 0.99 | 0.29 | 0.48 |
| TG continuous (mmol/L) | 0.99 (0.89-1.10) | 1.06 (0.99-1.13) | 0.99 (0.94-1.04) |
| *p* per 1 mmol/L increment | 0.85 | 0.12 | 0.65 |
| TC quartiles (mmol/L) |  |  |  |
| Q1(<4.27) | Ref. | Ref. | Ref. |
| Q2(4.27-4.91) | 1.27 (0.82-1.98) | 0.87 (0.65-1.16) | 1.19 (0.94-1.49) |
| Q3(4.91-5.57) | 1.13 (0.72-1.80) | 0.65 (0.47-0.89) | 1.36 (1.08-1.73) |
| Q4(≥5.57) | 1.34 (0.83-2.16) | 0.82 (0.60-1.13) | 1.47 (1.14-1.88) |
| *p*_trend_ | 0.60 | 0.07 | 0.02 |
| TC continuous (mmol/L) | 1.02 (0.91-1.14) | 0.96 (0.87-1.05) | 1.08 (1.02-1.16) |
| *p* per 1 mmol/L increment | 0.78 | 0.35 | 0.02 |
| LDL-C quartiles (mmol/L) |  |  |  |
| Q1(<1.84) | Ref. | Ref. | Ref. |
| Q2(1.84-2.34) | 1.08 (0.71-1.65) | 1.29 (0.96-1.74) | 0.82 (0.66-1.02) |
| Q3(2.34-2.83) | 1.29 (0.85-1.98) | 1.45 (1.09-1.92) | 0.86 (0.69-1.07) |
| Q4(≥2.83) | 0.82 (0.50-1.33) | 1.45 (1.09-1.92) | 0.67 (0.52-0.85) |
| *p*_trend_ | 0.16 | 0.08 | 0.01 |
| LDL-C continuous (mmol/L) | 0.94 (0.79-1.13) | 1.03 (0.92-1.15) | 0.84 (0.76-0.92) |
| *p* per 1 mmol/L increment | 0.52 | 0.64 | 0.0002 |
| HDL-C quartiles (mmol/L) |  |  |  |
| Q1(<1.28) | Ref. | Ref. | Ref. |
| Q2(1.28-1.51) | 1.08 (0.64-1.83) | 0.97 (0.72-1.32) | 0.75 (0.60-0.94) |
| Q3(1.51-1.77) | 1.84 (1.14-2.96) | 0.97 (0.71-1.32) | 0.76 (0.61-0.95) |
| Q4(≥1.77) | 2.50 (1.59-3.95) | 1.27 (0.94-1.72) | 0.91 (0.74-1.13) |
| *p*_trend_ | <.0001 | 0.20 | 0.02 |
| HDL-C continuous (mmol/L) | 2.24 (1.75-2.86) | 1.28 (1.00-1.65) | 1.00 (0.82-1.23) |
| *p* per 1 mmol/L increment | <.0001 | 0.05 | 1.00 |

Abbreviations: HR, hazard ratio; CI, hazard ratio; Q, quartile; TG, triglyceride; TC, total cholesterol; LDL-C, low-density lipoprotein cholesterol; HDL-C-C, high-density lipoprotein cholesterol.

^a^ In this model, deaths without GI malignancies diagnosis were calculated as competitive events using the proportional subdistribution hazards model, adjusted for age, gender, FBG, hs-CRP, BMI, WC, hypertension, physical activity, smoking status, drinking status, family cancer history, and other lipid biomarkers (e.g., TC, LDL-C, and HDL-C were adjusted in the analysis of TG).

**TABLE S2** Sensitivity Analysis I: Associations between lipid biomarkers (in quartiles and as continuous variables) and the risk of site-specific gastrointestinal cancers excluding participants who occurred GI malignancies within 1 year.

| Exposure | Esophagus cancer | Gastric cancer | Colorectal cancer |
| --- | --- | --- | --- |
|  | Adjusted HR^a^ (95%CI) | Adjusted HR^a^ (95%CI) | Adjusted HR^a^ (95%CI) |
| TG quartiles (mmol/L) |  |  |  |
| Q1 (<0.89) | Ref. | Ref. | Ref. |
| Q2 (0.89-1.26) | 1.03 (0.68-1.57) | 1.18 (0.87-1.61) | 1.13 (0.90-1.42) |
| Q3 (1.26-1.90) | 1.03 (0.67-1.59) | 1.09 (0.79-1.51) | 1.15 (0.91-1.45) |
| Q4 (≥1.90) | 1.09 (0.70-1.69) | 1.39 (1.01-1.92) | 1.02 (0.80-1.30) |
| *p*_trend_ | 0.99 | 0.11 | 0.50 |
| TG continuous (mmol/L) | 1.00 (0.90-1.11) | 1.06 (0.98-1.13) | 0.99 (0.93-1.05) |
| *p* per 1 mmol/L increment | 0.99 | 0.13 | 0.73 |
| TC quartiles (mmol/L) |  |  |  |
| Q1(<4.27) | Ref. | Ref. | Ref. |
| Q2(4.27-4.91) | 1.21 (0.77-1.89) | 0.90 (0.67-1.20) | 1.12 (0.88-1.42) |
| Q3(4.91-5.57) | 1.10 (0.69-1.75) | 0.67 (0.48-0.92) | 1.35 (1.07-1.72) |
| Q4(≥5.57) | 1.33 (0.83-2.13) | 0.82 (0.60-1.13) | 1.42 (1.10-1.83) |
| *p*_trend_ | 0.62 | 0.18 | 0.02 |
| TC continuous (mmol/L) | 1.02 (0.89-1.16) | 0.96 (0.87-1.06) | 1.07 (1.00-1.15) |
| *p* per 1 mmol/L increment | 0.83 | 0.42 | 0.06 |
| LDL-C quartiles (mmol/L) |  |  |  |
| Q1(<1.84) | Ref. | Ref. | Ref. |
| Q2(1.84-2.34) | 1.05 (0.68-1.62) | 1.35 (0.99-1.84) | 0.81 (0.65-1.02) |
| Q3(2.34-2.83) | 1.30 (0.86-1.97) | 1.50 (1.10-2.04) | 0.88 (0.70-1.10) |
| Q4(≥2.83) | 0.74 (0.45-1.21) | 1.28 (0.91-1.80) | 0.69 (0.54-0.88) |
| *p*_trend_ | 0.08 | 0.09 | 0.02 |
| LDL-C continuous (mmol/L) | 0.93 (0.77-1.12) | 1.04 (0.91-1.17) | 0.85 (0.77-0.94) |
| *p* per 1 mmol/L increment | 0.43 | 0.59 | 0.001 |
| HDL-C quartiles (mmol/L) |  |  |  |
| Q1(<1.28) | Ref. | Ref. | Ref. |
| Q2(1.28-1.51) | 1.34 (0.77-2.35) | 0.91 (0.67-1.24) | 0.75 (0.60-0.95) |
| Q3(1.51-1.77) | 2.26 (1.35-3.78) | 0.94 (0.69-1.28) | 0.78 (0.62-0.97) |
| Q4(≥1.77) | 3.00 (1.81-4.95) | 1.26 (0.93-1.71) | 0.90 (0.72-1.13) |
| *p*_trend_ | <.0001 | 0.08 | 0.04 |
| HDL-C continuous (mmol/L) | 2.33 (1.79-3.02) | 1.30 (1.01-1.66) | 1.00 (0.83-1.22) |
| *p* per 1 mmol/L increment | <.0001 | 0.04 | 0.97 |

Abbreviations: HR, hazard ratio; CI, hazard ratio; Q, quartile; TG, triglyceride; TC, total cholesterol; LDL-C, low-density lipoprotein cholesterol; HDL-C, high-density lipoprotein cholesterol.

^a^ Adjusted for age, gender, FBG, hs-CRP, BMI, WC, hypertension, physical activity, smoking status, drinking status, family cancer history, and other lipid biomarkers (e.g., TC, LDL-C, and HDL-C were adjusted in the analysis of TG).

**TABLE S3** Sensitivity Analysis II: Associations between lipid biomarkers (in quartiles and as continuous variables) and the risk of site-specific gastrointestinal cancers excluding participants under lipid-lowering therapy.

| Exposure | Esophagus cancer | Gastric cancer | Colorectal cancer |
| --- | --- | --- | --- |
|  | Adjusted HR^a^ (95%CI) | Adjusted HR^a^ (95%CI) | Adjusted HR^a^ (95%CI) |
| TG quartiles (mmol/L) |  |  |  |
| Q1 (<0.89) | Ref. | Ref. | Ref. |
| Q2 (0.89-1.26) | 1.05 (0.70-1.58) | 1.15 (0.85-1.56) | 1.17 (0.93-1.47) |
| Q3 (1.26-1.90) | 0.98 (0.64-1.51) | 1.10 (0.80-1.51) | 1.20 (0.96-1.52) |
| Q4 (≥1.90) | 1.05 (0.68-1.63) | 1.36 (0.99-1.87) | 1.08 (0.85-1.37) |
| *p*_trend_ | 0.98 | 0.26 | 0.37 |
| TG continuous (mmol/L) | 0.98 (0.87-1.09) | 1.06 (0.99-1.14) | 0.99 (0.94-1.05) |
| *p* per 1 mmol/L increment | 0.66 | 0.10 | 0.85 |
| TC quartiles (mmol/L) |  |  |  |
| Q1(<4.27) | Ref. | Ref. | Ref. |
| Q2(4.27-4.91) | 1.29 (0.83-2.02) | 0.84 (0.63-1.12) | 1.16 (0.92-1.47) |
| Q3(4.91-5.57) | 1.15 (0.73-1.82) | 0.63 (0.46-0.87) | 1.32 (1.04-1.68) |
| Q4(≥5.57) | 1.33 (0.83-2.13) | 0.79 (0.58-1.09) | 1.45 (1.13-1.87) |
| *p*_trend_ | 0.59 | 0.04 | 0.03 |
| TC continuous (mmol/L) | 1.02 (0.89-1.16) | 0.95 (0.86-1.04) | 1.08 (1.00-1.16) |
| *p* per 1 mmol/L increment | 0.83 | 0.24 | 0.04 |
| LDL-C quartiles (mmol/L) |  |  |  |
| Q1(<1.84) | Ref. | Ref. | Ref. |
| Q2(1.84-2.34) | 1.11 (0.73-1.71) | 1.29 (0.95-1.76) | 0.82 (0.66-1.02) |
| Q3(2.34-2.83) | 1.33 (0.88-2.02) | 1.48 (1.09-2.00) | 0.87 (0.70-1.08) |
| Q4(≥2.83) | 0.82 (0.51-1.34) | 1.26 (0.90-1.76) | 0.66 (0.51-0.84) |
| *p*_trend_ | 0.14 | 0.089 | 0.007 |
| LDL-C continuous (mmol/L) | 0.95 (0.79-1.14) | 1.04 (0.92-1.18) | 0.84 (0.76-0.92) |
| *p* per 1 mmol/L increment | 0.57 | 0.51 | 0.0003 |
| HDL-C quartiles (mmol/L) |  |  |  |
| Q1(<1.28) | Ref. | Ref. | Ref. |
| Q2(1.28-1.51) | 1.04 (0.61-1.77) | 1.02 (0.75-1.38) | 0.74 (0.59-0.92) |
| Q3(1.51-1.77) | 1.78 (1.10-2.88) | 0.97 (0.71-1.33) | 0.75 (0.60-0.93) |
| Q4(≥1.77) | 2.48 (1.56-3.95) | 1.30 (0.96-1.75) | 0.91 (0.73-1.13) |
| *p*_trend_ | <.0001 | 0.17 | 0.01 |
| HDL-C continuous (mmol/L) | 2.25 (1.73-2.93) | 1.29 (1.01-1.65) | 1.00 (0.83-1.21) |
| *p* per 1 mmol/L increment | <.0001 | 0.04 | 0.99 |

Abbreviations: HR, hazard ratio; CI, hazard ratio; Q, quartile; TG, triglyceride; TC, total cholesterol; LDL-C, low-density lipoprotein cholesterol; HDL-C, high-density lipoprotein cholesterol.

^a^ Adjusted for age, gender, FBG, hs-CRP, BMI, WC, hypertension, physical activity, smoking status, drinking status, family cancer history, and other lipid biomarkers (e.g., TC, LDL-C, and HDL-C were adjusted in the analysis of TG).

**TABLE S4** Sensitivity Analysis III: Associations between lipid biomarkers (in quartiles and as continuous variables) and the risk of site-specific gastrointestinal cancers excluding participants with high-fat dietary habit.

| Exposure | Esophagus cancer | Gastric cancer | Colorectal cancer |
| --- | --- | --- | --- |
|  | Adjusted HR^a^ (95%CI) | Adjusted HR^a^ (95%CI) | Adjusted HR^a^ (95%CI) |
| TG quartiles (mmol/L) |  |  |  |
| Q1 (<0.89) | Ref. | Ref. | Ref. |
| Q2 (0.89-1.26) | 1.09 (0.70-1.69) | 1.11 (0.81-1.52) | 1.13 (0.89-1.44) |
| Q3 (1.26-1.90) | 1.13 (0.72-1.77) | 1.07 (0.77-1.48) | 1.21 (0.95-1.53) |
| Q4 (≥1.90) | 1.04 (0.65-1.67) | 1.39 (1.01-1.92) | 1.07 (0.83-1.38) |
| *p*_trend_ | 0.96 | 0.16 | 0.45 |
| TG continuous (mmol/L) | 0.99 (0.88-1.10) | 1.06 (0.99-1.14) | 0.99 (0.93-1.05) |
| *p* per 1 mmol/L increment | 0.79 | 0.11 | 0.80 |
| TC quartiles (mmol/L) |  |  |  |
| Q1(<4.27) | Ref. | Ref. | Ref. |
| Q2(4.27-4.91) | 1.14 (0.71-1.82) | 0.89 (0.66-1.19) | 1.15 (0.90-1.46) |
| Q3(4.91-5.57) | 1.12 (0.70-1.80) | 0.66 (0.48-0.92) | 1.27 (0.99-1.63) |
| Q4(≥5.57) | 1.28 (0.79-2.08) | 0.82 (0.60-1.14) | 1.36 (1.05-1.77) |
| *p*_trend_ | 0.80 | 0.10 | 0.12 |
| TC continuous (mmol/L) | 0.97 (0.80-1.17) | 0.96 (0.87-1.05) | 1.05 (0.98-1.14) |
| *p* per 1 mmol/L increment | 0.75 | 0.37 | 0.17 |
| LDL-C quartiles (mmol/L) |  |  |  |
| Q1(<1.84) | Ref. | Ref. | Ref. |
| Q2(1.84-2.34) | 1.18 (0.75-1.85) | 1.25 (0.91-1.70) | 0.83 (0.66-1.03) |
| Q3(2.34-2.83) | 1.40 (0.91-2.17) | 1.49 (1.10-2.03) | 0.82 (0.65-1.04) |
| Q4(≥2.83) | 0.90 (0.54-1.49) | 1.23 (0.87-1.73) | 0.68 (0.53-0.88) |
| *p*_trend_ | 0.19 | 0.08 | 0.03 |
| LDL-C continuous (mmol/L) | 0.97 (0.80-1.17) | 1.05 (0.93-1.19) | 1.05 (1.04-1.06) |
| *p* per 1 mmol/L increment | 0.74 | 0.43 | 0.001 |
| HDL-C quartiles (mmol/L) |  |  |  |
| Q1(<1.28) | Ref. | Ref. | Ref. |
| Q2(1.28-1.51) | 0.99 (0.56-1.77) | 1.04 (0.76-1.42) | 0.75 (0.59-0.95) |
| Q3(1.51-1.77) | 1.91 (1.15-3.18) | 1.03 (0.75-1.41) | 0.79 (0.63-1.00) |
| Q4(≥1.77) | 2.55 (1.55-4.19) | 1.25 (0.91-1.71) | 0.87 (0.69-1.09) |
| *p*_trend_ | <.0001 | 0.44 | 0.08 |
| HDL-C continuous (mmol/L) | 2.30 (1.75-3.02) | 1.25 (0.97-1.61) | 0.96 (0.78-1.17) |
| *p* per 1 mmol/L increment | <.0001 | 0.08 | 0.66 |

Abbreviations: HR, hazard ratio; CI, hazard ratio; Q, quartile; TG, triglyceride; TC, total cholesterol; LDL-C, low-density lipoprotein cholesterol; HDL-C, high-density lipoprotein cholesterol.

^a^ Adjusted for age, gender, FBG, hs-CRP, BMI, WC, hypertension, physical activity, smoking status, drinking status, family cancer history, and other lipid biomarkers (e.g., TC, LDL-C, and HDL-C were adjusted in the analysis of TG).


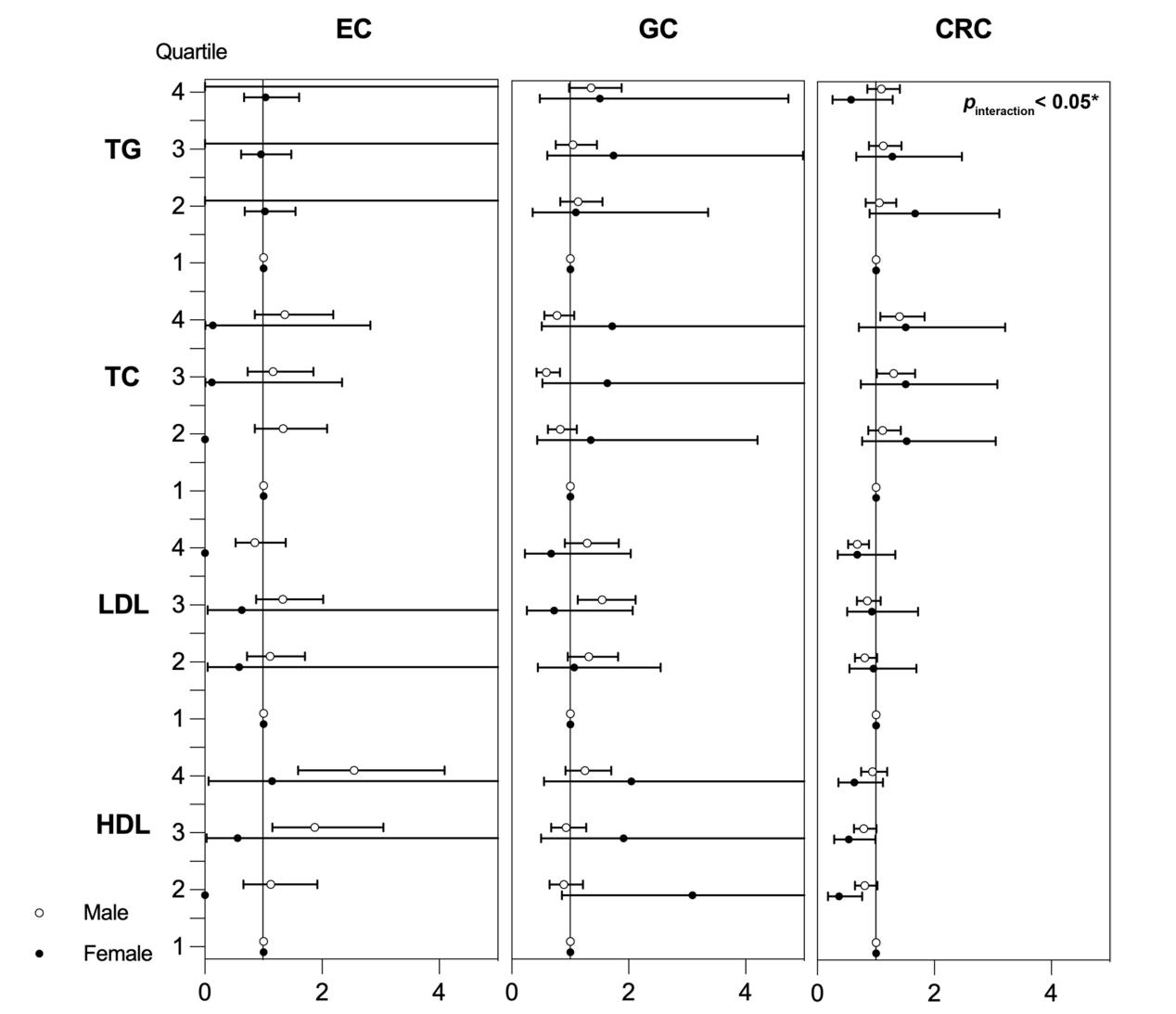


**FIGURE S1** Associations of lipid biomarkers in quartiles with the risk of site-specific gastrointestinal cancers by gender subgroups.

Abbreviations: HR, hazard ratio; CI, hazard ratio; TG, triglyceride; TC, total cholesterol; LDL-C, low-density lipoprotein cholesterol; HDL-C, high-density lipoprotein cholesterol.

^a^ Adjusted for age, FBG, hs-CRP, BMI, WC, hypertension, physical activity, smoking status, drinking status, family cancer history, and other lipid biomarkers (e.g., TC, LDL-C-C, and HDL-C-C were adjusted in the analysis of TG).

Statistically insignificant p for interactions in subgroup analysis were omitted.

* *p*_interaction_ < 0.05, statistically insignificant *p*_interaction_ in subgroup analysis was omitted.


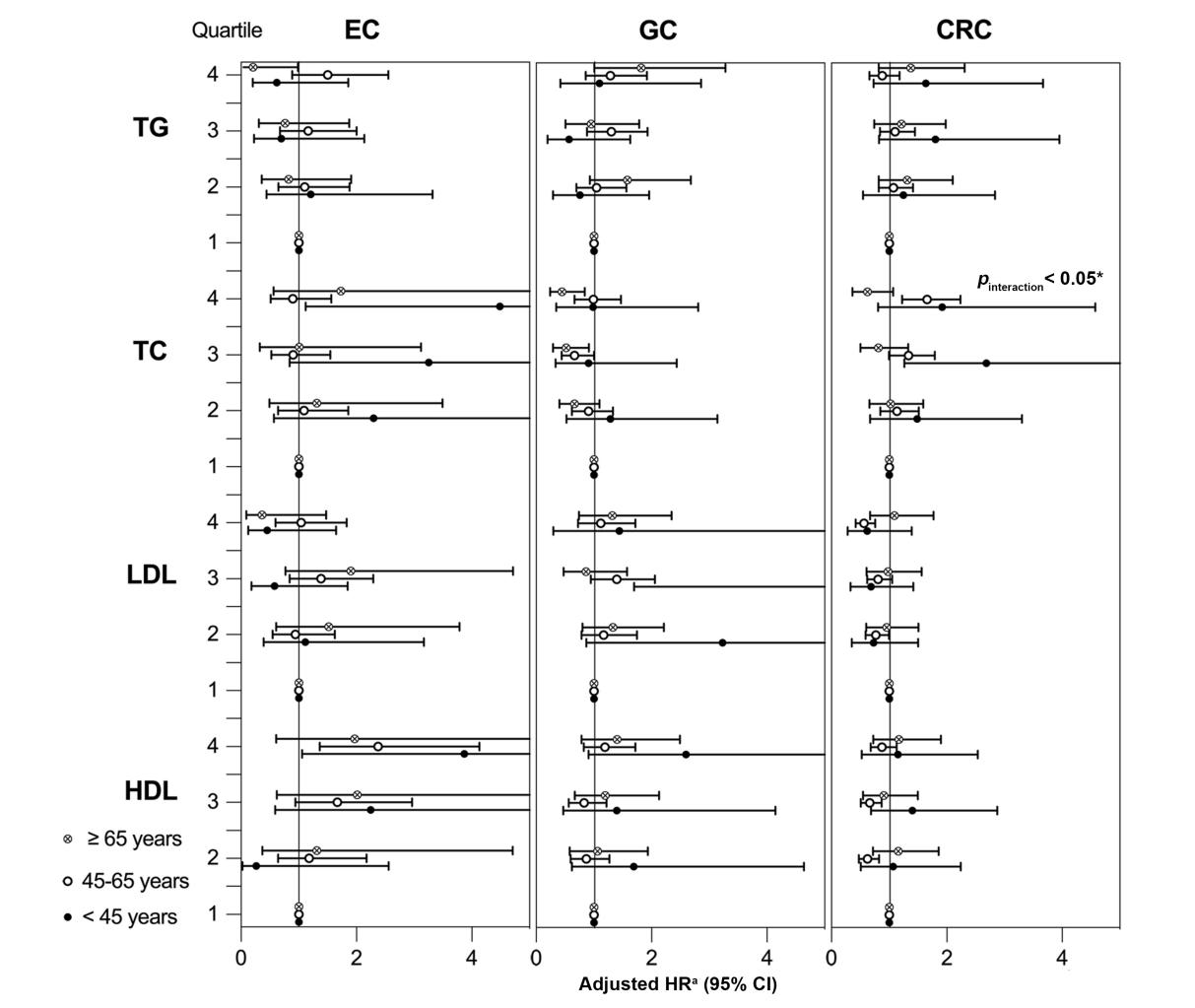


**FIGURE S2** Associations of lipid biomarkers in quartiles with the risk of site-specific gastrointestinal cancers by age subgroups.

Abbreviations: HR, hazard ratio; CI, hazard ratio; TG, triglyceride; TC, total cholesterol; LDL-C, low-density lipoprotein cholesterol; HDL-C, high-density lipoprotein cholesterol.

^a^ Adjusted for gender, FBG, hs-CRP, BMI, WC, hypertension, physical activity, smoking status, drinking status, family cancer history, and other lipid biomarkers (e.g., TC, LDL-C-C, and HDL-C-C were adjusted in the analysis of TG).

Statistically insignificant p for interactions in subgroup analysis were omitted.

* *p*_interaction_ < 0.05, statistically insignificant *p*_interaction_ in subgroup analysis was omitted.


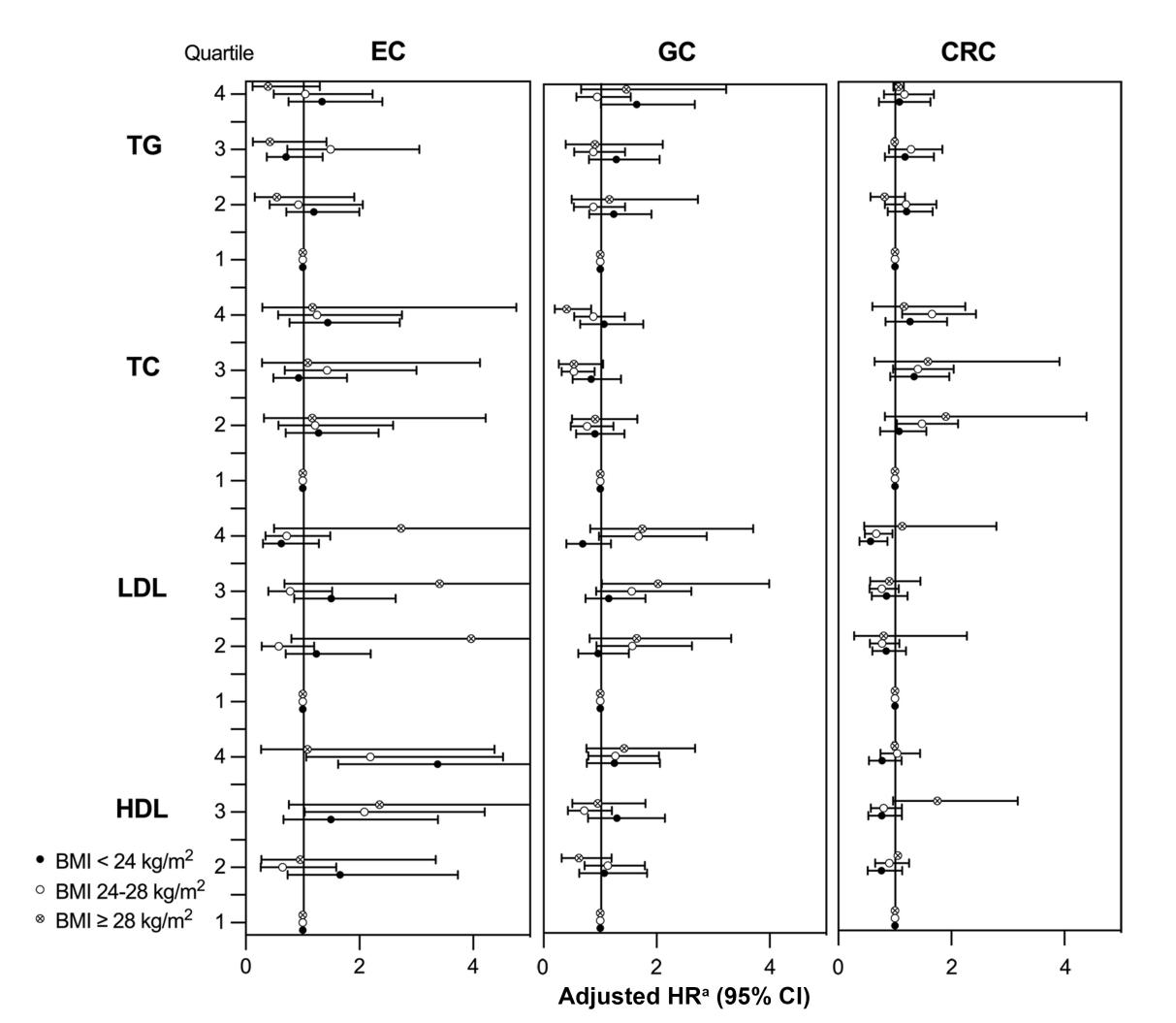


**FIGURE S3** Associations of lipid biomarkers in quartiles with the risk of site-specific gastrointestinal cancers by BMI subgroups.

Abbreviations: HR, hazard ratio; CI, hazard ratio; TG, triglyceride; TC, total cholesterol; LDL-C, low-density lipoprotein cholesterol; HDL-C, high-density lipoprotein cholesterol; BMI, body mass index.

^a^ Adjusted for age, gender, FBG, hs-CRP, WC, hypertension, physical activity, smoking status, drinking status, family cancer history, and other lipid biomarkers (e.g., TC, LDL-C-C, and HDL-C-C were adjusted in the analysis of TG).

* *p*_interaction_ < 0.05, statistically insignificant *p*_interaction_ in subgroup analysis was omitted.


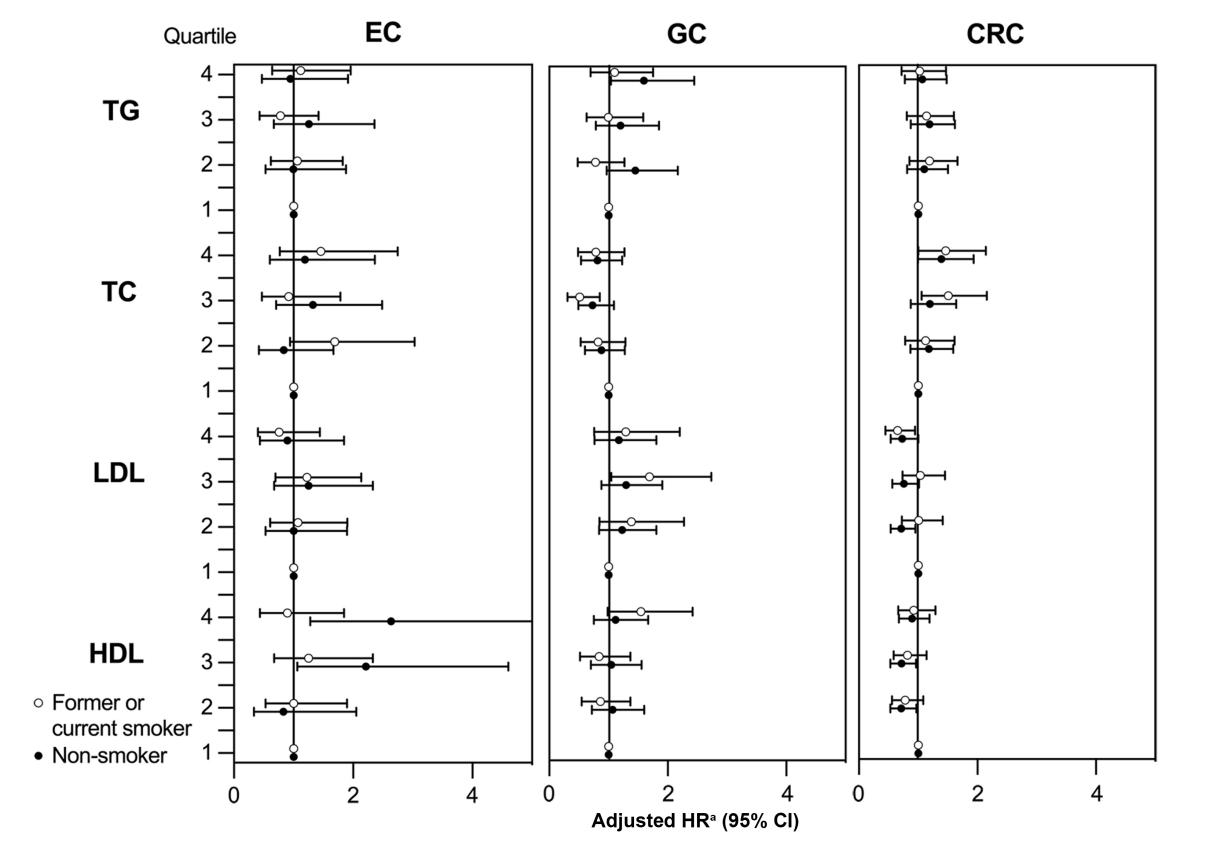


**FIGURE S4** Associations of lipid biomarkers in quartiles with the risk of site-specific gastrointestinal cancers by smoking status subgroups.

Abbreviations: HR, hazard ratio; CI, hazard ratio; TG, triglyceride; TC, total cholesterol; LDL-C, low-density lipoprotein cholesterol; HDL-C, high-density lipoprotein cholesterol.

^a^ Adjusted for age, gender, FBG, hs-CRP, BMI, WC, hypertension, physical activity, drinking status, family cancer history, and other lipid biomarkers (e.g., TC, LDL-C-C, and HDL-C-C were adjusted in the analysis of TG).

* *p*_interaction_ < 0.05, statistically insignificant *p*_interaction_ in subgroup analysis was omitted.


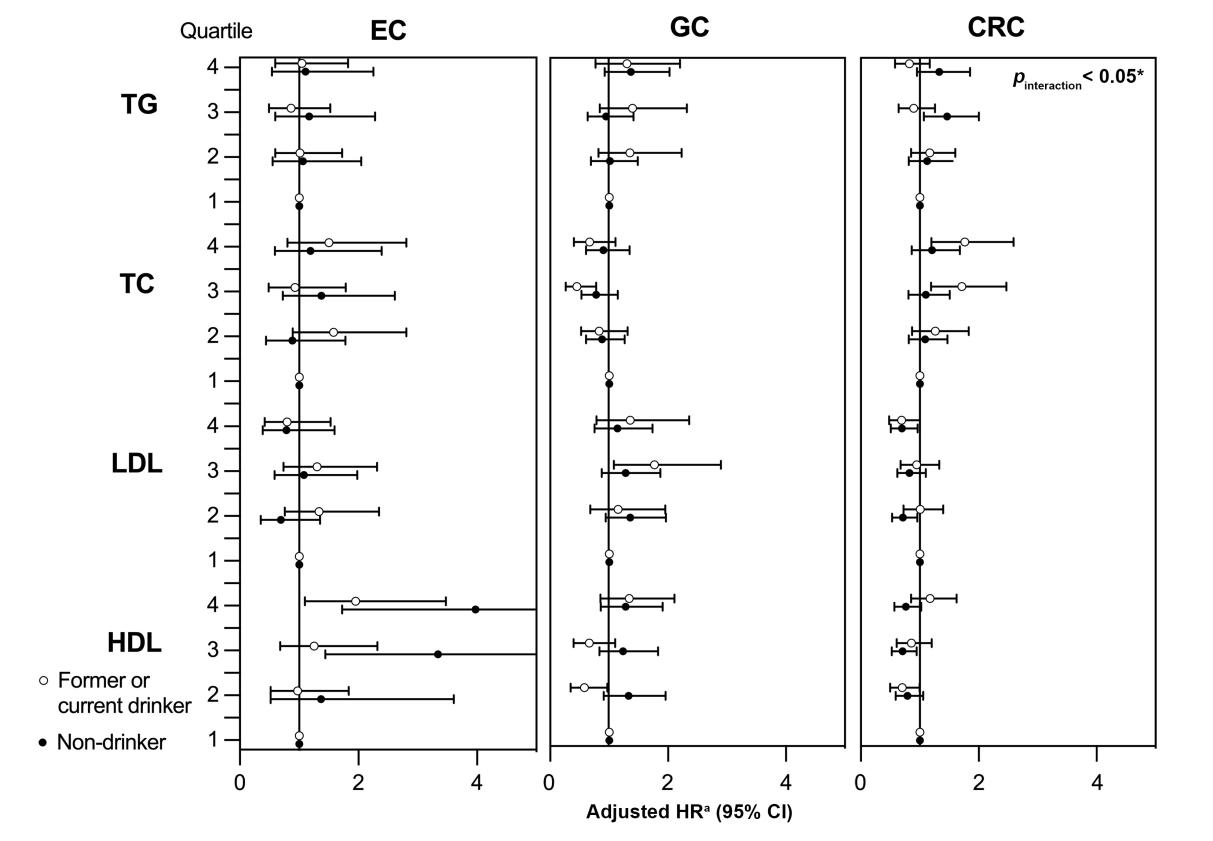


**FIGURE S5** Associations of lipid biomarkers in quartiles with the risk of site-specific gastrointestinal cancers by drinking status subgroups.

Abbreviations: HR, hazard ratio; CI, hazard ratio; TG, triglyceride; TC, total cholesterol; LDL-C, low-density lipoprotein cholesterol; HDL-C, high-density lipoprotein cholesterol.

^a^ Adjusted for age, gender, FBG, hs-CRP, BMI, WC, hypertension, physical activity, smoking status, family cancer history, and other lipid biomarkers (e.g., TC, LDL-C-C, and HDL-C-C were adjusted in the analysis of TG).

* *p*_interaction_ < 0.05, statistically insignificant *p*_interaction_ in subgroup analysis was omitted.
